# Changes in Present and Future Circulation Types Frequency in Northwest Iberian Peninsula

**DOI:** 10.1371/journal.pone.0016201

**Published:** 2011-01-21

**Authors:** María N. Lorenzo, Alexandre M. Ramos, Juan J. Taboada, Luis Gimeno

**Affiliations:** 1 EPhysLab, Facultad de Ciencias, Universidad de Vigo, Ourense, Spain; 2 Meteogalicia, Xunta de Galicia, Santiago de Compostela, Spain; University of Bristol, United Kingdom

## Abstract

The aim of the work described herein was to study projection scenarios in order to find changes in the synoptic variability of the northwest Iberian Peninsula in the 21st century. To this end, we investigated the changes in the frequency of the different circulation types computed for the study area using three different models used in the IPCC 4^th^ assessment report. The circulation types were computed using the procedure known as Lamb circulation types. The control simulation for the late 20th century was evaluated objectively from the results obtained using data from the NCEP/NCAR reanalysis, as to evaluate the ability of the model to reproduce the present climate. We have compared not only seasonal mean sea level pressure fields but also the mean seasonal frequency of circulation types. The results for the end of the 21st century show a decrease in the frequency of cyclonic, W, and SW circulation types in the spring and summer months. This trend also appears in the autumn, with a concomitant increase in the anticyclonic types.

## Introduction

The findings of recent studies have shown that the North Atlantic-European (NAE) sector is characterized by significant trends in climate that occur over different time scales [1]–[3]. The variability of the atmospheric circulation is the most important of these trends, with respect to changes in the spatial distribution and variability, not only for temperature and precipitation, but also for other climatological variables [4]–[7].

Circulation types reflect the atmospheric circulation that is specific to a given region, and may be obtained following the examination of synoptic daily data, usually on a regular grid, using any of a wide variety of methodologies [8], [9]. Over the last decade, there has been a growing interest among the scientific community in studying links between atmospheric processes and surface climate. Therefore, the classification of synoptic weather situations gained increased importance. Circulation types are used as a tool for a wide range of applications, such as climatological studies e.g. [5], [10], [11], [12], [13], biometeorology [14], air quality [15]–[17], and medium-range forecasting [18].

Changes in the frequency of occurrence of circulation types often result in changes in climatic variables at the earth's surface, such as precipitation e.g. [4], [19], [20] or temperature e.g [10], [13]. Furthermore, the frequency of the circulation types are sensible to the modes of the low-frequency variability and vice versa. In this way, changes in intensity or shifts in the position of the modes of low-frequency variability are related to changes in local circulation, i.e. circulation or circulation types [10], [21].

The use of circulation types can also be a useful tool for validating the control simulations of coupled general circulation models (CGCM), and assess the accuracy of the CGCMs, as well as for analyzing changes in circulation types under future scenarios of climate change. Despite this, very few papers compare the ability of GCMs to replicate historical circulation patterns. Hulme et al in 1993 [22] analyzed two control simulations of CGCM in order to evaluate if they are able to reproduce Lamb (1972) [23] circulation types in the UK. Demuzere et al in 2008 [24] carried out a similar study to analyze present and future ECHAM5 pressure fields using Lamb [23] circulation weather type for Belgium. Using a different approach, Schoof and Pryor in 2006 [25] used the Kirchhofer correlation-based map method to evaluate the ability of two GCMs in reproducing 500-hPa map pattern frequencies over the Midwestern US for the 1990–2001 period. A similar study was conducted by Anagnostopoulou et al in 2008 [26] where they studied the capability of the HadAM3P to reproduce the mean pattern and the frequency of circulation types concerning the 500 hPa geopotential height fields and the 1000–500 hPa thickness fields over Europe and the Mediterranean region. Huth in 2000 [27] tried a different approach by using a modification of the T-mode principal component analysis as a classification method in such a way that this classification procedure was applied to reanalysis [28] daily data 500 hPa geopotential height patterns and to those simulated by the control ECHAM3 GCM runs.

The main purpose of the study described herein was to investigate changes in the frequency of Lamb [23] circulation weather that affect the northwest (NW) Iberian Peninsula not only in the present climate but also in future climate change scenarios, by means of the output from several of the CGCMs used throughout the IPCC Fourth Assessment Report (4AR).

## Materials and Methods

As previously mentioned, the circulation types used in this work were computed using the procedure specified by Lamb [23], [29] in which the method was applied to the NW Iberian Peninsula (Galician area). In our study, we used two different SLP datasets in order to compute the circulation types:

a) The National Center for Environmental Prediction/National Center for Atmospheric Research (NCEP/NCAR) reanalysis data [28] (2.5°×2.5° longitude-latitude) of the daily mean SLP for the January 1948 - December 2008 period (http://www.cdc.noaa.gov/cdc/data.ncep.reanalysis.html). The use of this period of time allows not only the study of recent trends in circulation type but also the comparison to the output of the CGCMs models for the present climate (1961–1999 period). In doing so, we are evaluating them.

b) Data from three models that were studied in the IPCC 4AR, namely the IPSL climate system model IPSL-CM4 from Institut Pierre Simon Laplace des Sciences de l'Environment Global (IPSL), France, with a spatial resolution of 2.5° by 3.75°; the 5^th^ generation of the ECHAM general circulation model (ECHAM5/MPI-OM) from the Max Planck Institute for Meteorology, Hamburg, Germany, with a spatial resolution of ∼1.9° by 1.9°, and finally the CCSM3, NCAR Community Climate System Model 3.0 from the USA, with a spatial resolution of 1.4° by 1.4°. Readers who require more information about the salient features of these CGCMs should refer to Table 8.1 of [1]. The choice of these models was determined mainly by the availability of daily data, but the use of other GCMs simulations would be important and more reliable when it comes to quantifying uncertainties. Data of daily sea level pressure (SLP) of the three climate models and from three different forcing simulations were obtained from the WCRP CMIP3 multi-model database (https://esg.llnl.gov). These simulations correspond to three emission scenarios from the special report on emission scenarios (SRES) of low (B1), medium (A1B), and high (A2) concentrations of greenhouse gas [1]. The climatological normal period used in the 20th century is defined as the 1961–1999 period. For the 21st century, the coincidental temporal resolution availability of daily data for the 3 models allows us to analyze two periods, the first between 2046 and 2065 and the second between 2081 and 2100.

In order to compute the circulation types we need SLP daily data at a 2.5°×2.5° longitude-latitude regular grid. The points under consideration are located between 35 - 55°N and 25°W - 5°E. The circulation conditions were determined using physical or geometrical parameters, such as the direction and strength of airflow, and degree of cyclonicity obtained using 16 grid points. We considered only 10 types of circulation: eight driven by the direction of the flow (NE, E, SE, S, SW, W, NW, N) and two by the shear vorticity (cyclonic C or anticyclonic A). This method was adopted successfully for the NW Iberian Peninsula (Galician area) by Lorenzo et al in 2008 [10], who also provided a comprehensive description.

Since the CGCMs daily SLP data are at different spatial scales we interpolated (by means of a bicubic interpolation) the SLP fields of the different CGCMs simulations in order to obtain a set of regular 2.5°×2.5° grids that were then used to compute the circulation types for the different CGCMs and the different time periods.

The investigating design was as follows:

a) computation of the circulation types for the NW Iberian Peninsula for the 1948–2008 period using the NCEP/NCAR reanalysis;

b) evaluation of the GCMs SLP mean seasonal fields by comparing it to the NCEP/NCAR reanalysis SLP fields for the present climate (1961–1999 period);

c) the ability of the GCMs simulations to reproduce the mean frequency of the circulation types for the present climate was also compared objectively to the results obtained when using the NCEP/NCAR reanalysis.

d) removal of the systematic seasonal errors that were observed in the SLP mean fields of each model. To this purpose we computed, for each grid point, the seasonal mean bias as the difference between the mean seasonal SLP values obtained from the CGCMs and those obtained from the NCEP/NCAR data. The new daily fields of the CGCMs were then computed by removing the corresponding grid point seasonal bias for each model in the daily fields.

e) computation of the mean frequency of the circulation types using the new CGCMs daily adjusted SLP fields for the present climate and compare it again to the results obtained when using the NCEP/NCAR reanalysis in order to see if there is any improvement in the quality of the GCM SLP fields.

f) computation of the mean frequency of the circulation types for the CGCMs future scenarios (2046–2065 and 2081–2100 periods) and comparison with the mean frequency of the circulation types obtained using the present simulations of the CGCMs.

The Mann-Kendall trend test [30]–[32] was applied as to analyze the significance of the trends. This non-parametric test uses a correlation between the ranks of a time series and their time order and it is widely applied to time series of environmental data [33].

## Results

### 1. Frequency and trends in circulation type in the NW Iberian Peninsula during the 20^th^ century

**Figure 1:**
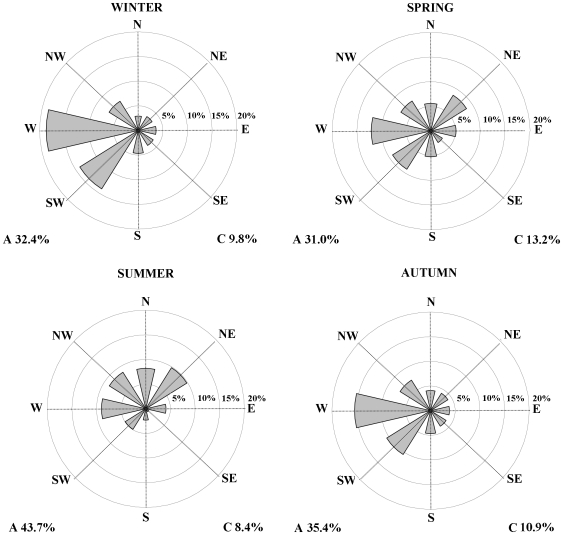
Mean frequency (%) of circulation type in winter (DJF), spring (MAM), summer (JJA), and autumn (SON). These frequencies were computed with the NCEP/NCAR reanalysis SLP fields for the 1948-2008 period.

The Jones et al in 1993 [29] procedure was adopted, thereby maintaining the 10 main circulation types (NE, E, SE, S, SW, W, NW, N, Anticyclonic (A) and Cyclonic (C)) for the NW Iberian Peninsula, relative to the 1948–2008 period, using the NCEP/NCAR reanalysis dataset. The relative frequency of each synoptic circulation type for each season is shown in Figure 1. It is interesting to note the changes in the direction of the flow between winter and summer. In winter, the flow comes mainly from the W and SW, with a large proportion of type A (32%) and type C (10%) circulation, but in summer the flow comes mainly from the Northern regions (NW, N and NE) but also from the W (8%), with a large proportion of type A circulation (44%). The autumn circulation types frequency are very similar to the ones found in the winter months, whereas the spring months appear to be characterized by a mixture between the circulation types frequency found in the summer and winter seasons. Type A varies between 43.7% in summer and 31% in spring, whereas type C varies between 13.2% in spring and 8.4% in summer.

The mean frequency of the circulation types in each season for the 1948–2008 period was also computed. Linear trends were obtained, taking into account not only the entire period of analysis but also the two sub-periods: 1948–1975 and 1976–2008. The year 1975 coincides approximately with a general change in circulation in the Northern Hemisphere [34], [35]. The Mann-Kendall test was applied to analyze the significance of the trends.

Only those results that are significant at least at the 5% level are shown in Table 1. One can see that for the entire 1948–2008 period, significant trends are only observed in winter and summer. Winter is characterized by a decrease in the frequency of the NW type, whereas in summer there is an increase in the frequency of the SW and C types.

**Table 1:** Linear trends (percentage/year) for the different circulation types computed with the NCEP/NCAR reanalysis sea level pressure field for the three periods studied being shown only results that are significant (Mann-Kendall rank test) at **0.01 or *0.05 level are shown.

	1948–2008	1948–1975	1976–2008
**Winter**	NW *-0.059		C *-0.187
			A *0.361
**Spring**			SW *0.113
			N**-0.154
**Summer**	SW ** 0.060	N *0.144	
	C *0.083		
**Autumn**		N **0.114	NE *0.1633

When treated separately, the significant trends in each period are higher, particularly in the second period. In summer and autumn an increase in the N circulation type for the first period (1948–1975) may be observed, although this trend does not continue into the second period after 1975.

While in the first period (1948–1975), no trends can be observed for types C and A, in the second period (1976–2008) there is a clear increase in type A and a decrease in type C for the winter months. Analysis of the second period shows an increase in the frequency of the SW and a decrease in the frequency of the N circulation types in spring. This could be related to the observed upward trend in maximum and minimum temperatures in this period, especially in spring, where the maximum temperature values increase at an average rate of about 1.0°C per decade [3]. Finally, an increase in the frequency of the NE type is again observed in autumn in the second period.

### 2. Evaluation and intercomparation between the NCEP/NCAR reanalysis data and model data

#### 2.1 Evaluation of GCMs SLP fields using NCEP/NCAR reanalysis data in the present climate

**Figure 2:**
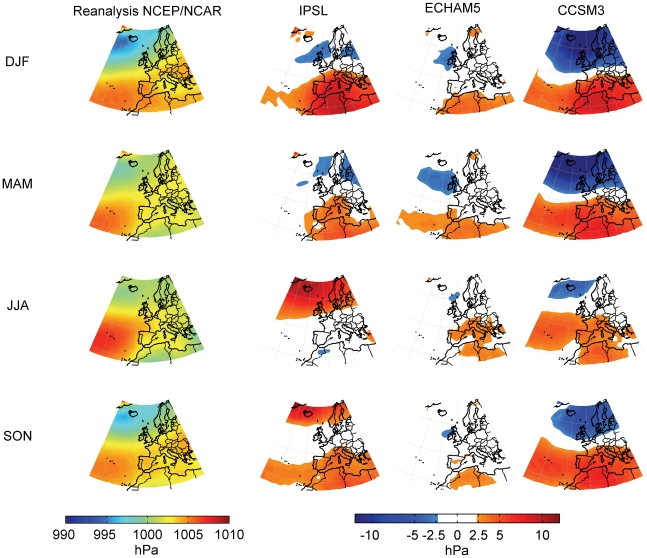
Mean seasonal sea level pressure differences for the 1961-1999 period. First column shows the seasonal mean sea level pressure during the 1961-1999 period using the NCEP/NCAR reanalysis data. The next columns show the difference between the mean sea level pressure from the three models (IPSL, ECHAM5, CCSM3) and the mean sea level pressure from the NCEP/NCAR reanalysis during the period 1961-1999. Season were defined as follow: winter (DJF), spring (MAM), summer (JJA), and autumn (SON).

Before analyzing the changes in the frequency of the circulation types for the 21^st^ century, the SLP mean fields of the three models were evaluated using the NCEP/NCAR reanalysis data obtained between 1961 and 1999 for each season (DJF, MAM, JJA and SON). In Figure 2, the seasonal mean SLP fields obtained from the three models and from the reanalysis are presented, as well as the difference between the two datasets. It is generally the case that the CCSM3 model produces the highest discrepancy in all four seasons. This model reproduces values of SLP that are too low at high latitudes and too high at mid latitudes, which leads to strong modeled westerlies over the North Atlantic. The IPSL reproduces values of SLP that are too high in the Mediterranean in winter, spring, and autumn, while in the summer there is a high SLP anomaly in the Iceland region. The ECHAM5 model reproduces patterns of SLP that are closest to the reanalysis data.

#### 2.2 Evaluation of the circulation types frequency of GCMs by comparing it to the NCEP/NCAR reanalysis data in the present climate

After evaluating the SLP mean fields produced by the three models using the NCEP/NCAR reanalysis data, we computed the circulation types for the control simulations for the late 20^th^ century and for the 21^st^ century, using the three CGCMs and applying the same methodology as described in section 3.

The frequency of the circulation types in the CGCMs was then compared objectively to the ones obtained previously using the reanalysis data of NCEP/NCAR in the 1961–1999 period. As mention in the Data and Methodology section, we interpolated the SLP fields of the CGCMs to obtain a set of regular 2.5°×2.5° grids that were then used to compute the circulation types. Table 2, Table 3, Table 4 and Table 5 (Original SLP data columns) show the percentage differences in frequency between the results obtained from the CGCMs and those obtained from the NCEP/NCAR reanalysis data for the different seasons: winter (Table 2), spring (Table 3), summer (Table 4) and autumn (Table 5). In bold are shown the differences that are significant at level 0.05 using a Wilcoxon rank sum test. The results show that the ECHAM5 model provides the best agreement when compared to the reanalysis data, showing differences of less than 5% in frequency per type, except in summer, where the model shows a higher frequency than the actual one for type A by 10%. This result is not unexpected, because the ECHAM5 model is the one that offers the lowest differences in the mean field (Figure 2). On the other hand, the CCSM3 model produces the highest frequency bias in the majority of cases, with differences of up to 20% in the frequency of the W type. This disagreement could be explained by the fact that this model overestimates the magnitude of the westerlies at the mid-latitudes ([36] and Figure 2). The IPSL model shows the greatest differences in summer, when the high positive anomalies of SLP at high latitudes leads to a decrease in the frequency of type A and an increase in the frequency of type NE over the NW of the Iberian Peninsula. For winter, this model shows a decrease in the frequency of type A against an increase in the frequency of types SW and W, the reason for which is connected to the anomalies in SLP seen in Figure 2, in which it is possible to observe an eastward displacement of high and low pressures at high latitudes.

**Table 2:** Differences (%) in mean frequency of the circulation types for the winter each season between the output of the three models of the IPCC 4AR (IPSL, ECHAM5 and CCSM3) and the NCEP/NCAR reanalysis data, for the 1961–1999 period when using the original sea level pressure (SLP) data and the corrected sea level pressure (SLP) data.

Winter	ORIGINAL SLP DATA			CORRECTED SLP DATA		
WT	IPSL	ECHAM5	CCSM3	IPSL	ECHAM5	CCSM3
**NE**	**−2.71**	**−1.32**	**−2.74**	**−1.38**	**−**0.14	**−**0.77
**E**	**−2.36**	**−1.35**	**−3.32**	**−**0.69	**−**0.76	**−**0.83
**SE**	**−2.58**	**−**1.28	**−3.17**	**−**1.41	**−**0.24	**−1.68**
**S**	**−1.84**	**−**0.88	**−3.53**	**−**0.08	0.21	**−1.00**
**SW**	**13.25**	2.83	**3.95**	0.94	**−**0.54	**−**1.88
**W**	**10.83**	**5.92**	**22.10**	0.70	**−**0.27	0.76
**NW**	**−**1.20	**2.06**	1.78	1.41	1.44	**3.72**
**N**	**−1.63**	0.32	**−1.47**	0.74	**1.03**	0.91
**C**	**−**2.65	**3.19**	**−4.81**	0.52	1.90	**−**1.13
**A**	**−9.12**	**−9.50**	**−8.79**	**−**0.75	**−**2.63	1.90

In bold are shown the differences which are significant at level 0.05 using a Wilcoxon rank sum test.

**Table 3:** The same as Table 2 but for spring season.

Spring	ORIGINAL SLP DATA			CORRECTED SLP DATA		
WT	IPSL	ECHAM5	CCSM3	IPSL	ECHAM5	CCSM3
**NE**	**−3.53**	**−4.63**	**−6.97**	0.44	**−**1.39	**−**1.56
**E**	**−**1.63	**−2.32**	**−4.54**	0.37	1.05	**−**0.08
**SE**	**−**0.13	0.01	**−1.74**	0.43	0.67	**−**0.18
**S**	**−**1.37	**−**1.58	**−4.26**	**−**1.17	0.62	**−**0.80
**SW**	**3.38**	**5.37**	**4.11**	0.12	1.17	**−**0.01
**W**	**5.93**	**8.36**	**21.15**	0.48	**−**1.49	3.33
**NW**	**−**1.07	1.21	**1.97**	**−**1.03	**−**0.62	**1.80**
**N**	**−**0.71	**−1.89**	**−3.88**	0.88	**−**0.97	**−**1.04
**C**	**−**2.13	0.41	**−7.76**	**−**1.23	0.56	**−**0.69
**A**	1.26	**−4.94**	1.93	0.70	0.40	**−**0.77

In bold are shown the differences which are significant at level 0.05 using a Wilcoxon rank sum test.

**Table 4:** The same as Table 2 but for summer season.

Summer	ORIGINAL SLP DATA			CORRECTED SLP DATA		
WT	IPSL	ECHAM5	CCSM3	IPSL	ECHAM5	CCSM3
**NE**	**13.32**	**−6.14**	**−3.68**	1.94	**−**1.38	**−**1.92
**E**	**−2.52**	**−1.69**	**−**1.31	**−2.52**	**−**0.18	**−**0.05
**SE**	**−0.47**	**0.76**	0.00	**−**0.31	**1.15**	**0.53**
**S**	**−1.40**	**−**0.44	**−1.04**	**−1.24**	0.27	**−**0.19
**SW**	**−2.79**	0.13	**−2.74**	**−**0.87	**−**0.52	**−1.78**
**W**	**−5.29**	**3.02**	0.02	**−2.41**	**−**0.46	0.81
**NW**	**−**0.27	1.06	1.09	0.45	1.33	1.93
**N**	**11.83**	**−3.89**	**−**1.69	**5.16**	**−1.63**	**−**0.66
**C**	**3.44**	**−3.59**	**−3.36**	**−**0.87	**−**1.19	0.08
**A**	**−15.86**	**10.78**	**12.72**	0.68	2.61	1.25

In bold are shown the differences which are significant at level 0.05 using a Wilcoxon rank sum test.

**Table 5:** The same as Table 2 but for autumn season.

Autumn	ORIGINAL SLP DATA			CORRECTED SLP DATA		
WT	IPSL	ECHAM5	CCSM3	IPSL	ECHAM5	CCSM3
**NE**	1.25	**−**1.39	**−2.85**	**5.21**	0.90	0.34
**E**	**−**1.22	**−1.36**	**−3.16**	0.35	**−**0.21	**−**0.04
**SE**	**−2.39**	**−1.67**	**−2.98**	**−2.13**	**−**0.83	**−**1.04
**S**	**−1.95**	**−**0.86	**−3.22**	**−**0.70	0.35	0.26
**SW**	**4.99**	2.34	**2.65**	1.23	0.67	1.00
**W**	**7.24**	3.09	**14.33**	0.41	**−**0.15	**−**0.81
**NW**	**−**0.10	**2.05**	**3.54**	**−**1.54	**2.32**	1.09
**N**	0.25	**−**0.23	**−1.50**	0.19	0.27	**−**0.48
**C**	**−**1.03	**−**1.54	**−5.82**	**−3.16**	1.78	**−**1.61
**A**	**−7.04**	**−**0.44	**−**1.01	0.15	**−5.10**	1.30

In bold are shown the differences which are significant at level 0.05 using a Wilcoxon rank sum test.

The classification of circulation types obtained herein, which makes use of the Lamb types [23], is performed on the SLP field. To this purpose, we computed the circulation types for each model again, this time without the corresponding systematic seasonal errors that were observed in the SLP field of each model, following the procedure of Demuzere et al in 2008 [24] and described in the Data and Methodology section.

The results obtained using this correction show the improvement that can be achieved by using this method. In Table 2, Table 3, Table 4, Table 5 (Corrected SLP data columns), we show the differences in the mean frequency of the circulation types for each season between the three models and the NCEP/NCAR reanalysis data for the 1961–1999 period when the corrected SLP data is used. A comparison of the results presented in Table 2, Table 3, Table 4 and Table 5, that is the original SLP data vs the corrected SLP data columns, shows that the difference between the circulation types frequency obtained for the CGCMs and the reanalysis is rather lower when the correction is applied, the differences being less than 5% in the majority of cases. In bold are printed the differences which are significant at level 0.05 using a Wilcoxon rank sum test. Therefore, it is possible to conclude that the differences in the circulation types frequency between the models and NCEP/NCAR reanalysis are mainly due to the seasonal mean bias.

### 3. Changes in the frequency of the circulation types for the 21^st^ century

The changes in the frequency of the circulation types for each of the three scenarios A1B, B1 and A2 were computed, and the differences between the respective control run and the two periods 2046–2065 and 2081–2100 using all three models were obtained.

**Figure 3:**
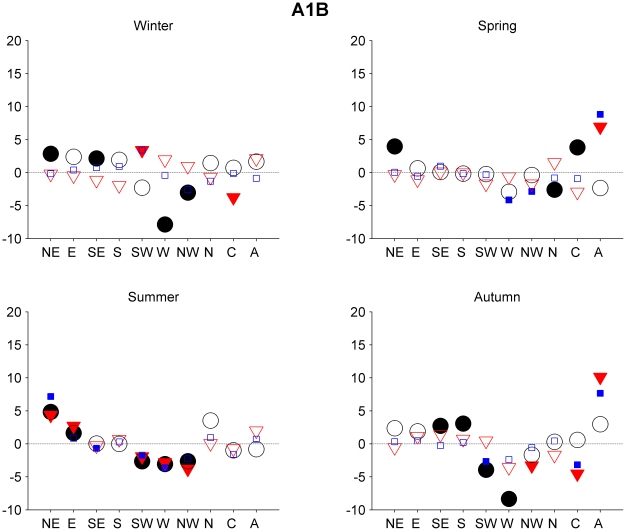
Projected frequency of the circulation types changes for the late 21st century SRES A1B scenario. Projected seasonal mean frequency of the circulation types changes (%) for the late 21st century (2081-2100) in the scenario A1B. The mean frequency of the circulation types are relative to the 1961-1999 period (IPSL, black circle, ECHAM5, red triangle and CCSM3, blue square). The filled symbols show the significant differences at level 0.05 after applying a Wilcoxon rank sum test. Season were defined as follow: winter (DJF), spring (MAM), summer (JJA), and autumn (SON).

**Figure 4:**
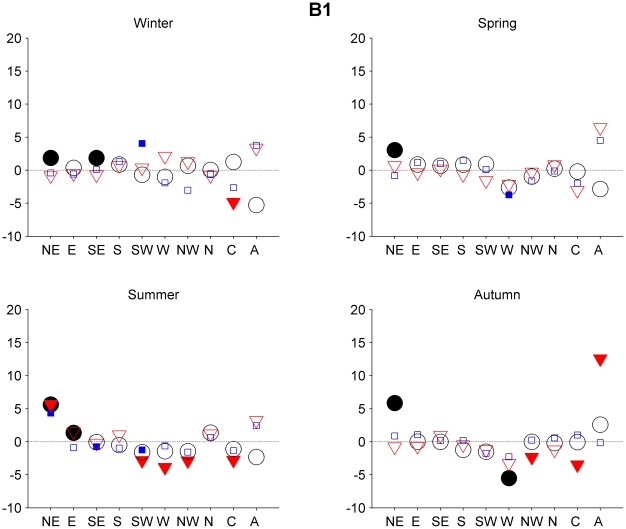
Projected frequency of the circulation types changes for the late 21st century SRES B1 scenario. The same as Figure 3 but for the SRES B1 scenario.

**Figure 5:**
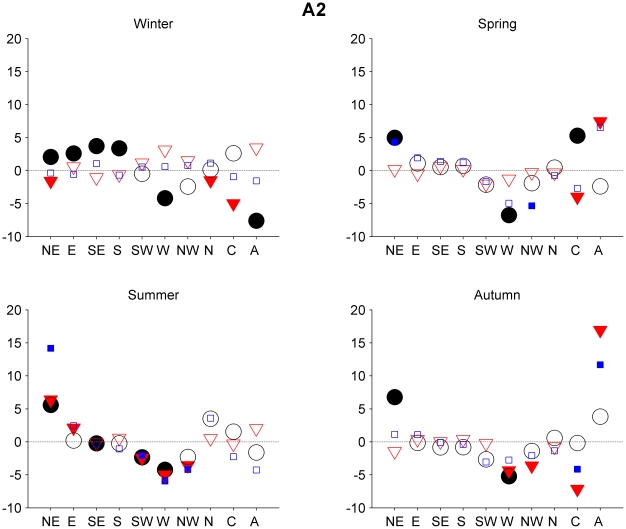
Projected frequency of the circulation types changes for the late 21st century SRES A2 scenario. The same as Figure 3 but for the SRES A2 scenario.

For all the models, the major changes can be found between the control run, 1961–1999, and the 2081–2100 period. Regarding the 2046–2065 period, there are no differences in the frequency greater than 6%. That being the case, we decided to focus our attention on the second period. The differences regarding the second period (2081–2100) are greater, particularly in scenarios A1B and A2. Figure 3, Figure 4 and Figure 5 show changes in the frequency of the circulation types during the 2081–2100 period with respect to those for the 1961–1999 period for the three different SRES scenarios. We focus here only on those changes that are significant at level 0.05 or greater.

Scenario A1B (Figure 3) leads to significant changes in all seasons; in winter, the frequency of type C decreases in the ECHAM5 and CCSM3 models. This would result in a decrease in precipitation in the domain, due to the fact that type C is one of the most important in explaining winter rainfall over the NW of the Iberian Peninsula [10]. In spring, there is an increase in the frequency of type A in the ECHAM5 and CCSM3 models. In summer, all models show an increase in the frequency of type NE and a decrease in the frequency of types SW and W. Furthermore, in two out of the three models, an increase is observed in the frequency of type E, along with a decrease in the frequency of the NW types. The picture as a whole shows a general intensification of the easterlies compared with the westerlies. Finally, under this scenario, the autumn results show a significant decrease in the frequency of types SW, W, and C, and a significant increase of type A, which would result in a decrease in precipitation, because all these types are linked with precipitation in the region [3].

Under scenario B1 (Figure 4), in general, no significant changes can be seen in relation to the 1961–1999 period. It is only in summer that we observe a significant change for the three models (α = 0.05), with an increase in the frequency of the NE types and a decrease in the frequency of the SW types in both ECHAM5 and CCSM3.

As expected, the scenario A2 (Figure 5) is associated with the greatest set of changes. In this case, the models (IPSL in particular) seem to show different behavior in winter and spring. The CCSM3 and ECHAM5 models show an increase in the frequency of type A and a decrease in the frequency of type C. The three models are in agreement in terms of a projected decrease in type C in spring. In summer, all three models also project an intensification of the easterlies, with a general increase in the frequency of the NE types and a decrease in the frequency of the SW, W, and NW types. Finally, in autumn season there is a decrease in the frequency of types W and C and an increase in the frequency of type A, as projected by at least two of the models.

The general trend describes a decrease in the C and SE types in spring, summer and autumn. For the autumn, we observe a significant increase in the A type. Furthermore, in summer, the changes in frequency of different circulation types show an intensification of the easterlies in relation to the westerlies. In winter, the frequency of type C decreases and the frequency of type A increases.

## Discussion

In the last years, few works have studied the ability of CGCMs in reproducing circulation patterns. In this respect, in this paper the procedure known as Lamb circulation types [23], [29] is used to evaluate the ability of CGCMs to reproduce the present mean frequency of the circulation types over the NW of the Iberian Peninsula and to study the projection scenarios as to find changes in the circulation types variability in the 21^st^ century. In this work we also considered the procedure of Demuzere et al in 2008 [24] to compute the circulation types for each GCMs, that is, without the corresponding systematic seasonal errors that are observed in the SLP field of each model for the present climate. The obtained results present an important improvement when the systematic errors are removed from the SLP fields, since the differences in the circulation types frequency between the models and the reanalysis are lower when compared to the results without the SLP corrections. Therefore, we can conclude that in most cases the discrepancies in the circulation types between the models and the reanalysis can be explained by the seasonal mean bias. On the other hand, the frequency of existence of different circulation types in a given area gives valuable information as to assess climate change. In this work we have used the results of three different GCMs under the three standard scenarios from the IPCC (A2, A1B and B1). We have used a Lamb-type classification, following the procedure used in a previous work, of which we present a catalogue of circulation types for the NW Iberian Peninsula.

First, trends in circulation type in the NW Iberian Peninsula were analyzed during the 20^th^ Century. In the second period (1976–2008) of the analysis there is a clear increase in type A and a decrease in type C for the winter months, suggesting a relationship between the positive phase of the NAO [10] and its eastward displacement towards Europe that has taken place over the last 20 years [21], [37].

We carried out an evaluation of GCMs SLP fields using NCEP/NCAR reanalysis data in the present climate in order to study if the GCMs are able to reproduce the seasonal mean SLP fields. The ECHAM5 model produces patterns of SLP that are the closest to the reanalysis data. Previous comparisons of the output of this model with the reanalysis ERA-40 [36] confirm that the use of ECHAM5 can yield realistic SLP fields, while the ability of the IPSL and the CCSM3 models to represent the SLP fields is rather poor.

Moreover, we computed the circulation type for each model again without the corresponding systematic seasonal errors that was observed in the SLP field of each model, following the procedure of Demuzere et al in 2008 [24]. The obtained results using this correction show some improvement, seen that the differences between the models and the reanalysis circulation types mean frequency are lower than the results without the SLP corrections.

Finally, changes in the mean circulation types frequency for the 21^st^ century were analyzed. Small changes (most of them less than 6% significant) in the frequency of the types were found for the 2046–2065 period, while the differences in the second period (2081–2100) are greater, particularly in scenarios A1B and A2. In summary, for the second period, results for all scenarios seem to show a decrease for types Cyclonic, W, and SW in spring and summer. This change is larger for scenario A2. Based on the relationship between the different circulation types and precipitation in Galicia region for present climate [3], [10] one can make the assumption that there will be dry conditions in these seasons. These results are in agreement with previous studies of precipitation in future scenarios. [38], [39]. For autumn we may also assume dryer conditions, based on the expected decrease in types W and Cyclonic and an increase in the Anticyclonic type.

This behavior is in agreement with the increase in NAO index expected toward the end of the 21th century for those scenarios [40]. Moreover, in summer, changes in frequency of different circulation types show an intensification of the easterlies over the westerlies. This has an important consequence for the area under study, because easterlies enhance upwelling of coastal waters, improving primary productivity in the area [41], [42].
